# The Role of the Lateral Hypothalamus in Violent Intraspecific Aggression—The Glucocorticoid Deficit Hypothesis

**DOI:** 10.3389/fnsys.2018.00026

**Published:** 2018-06-08

**Authors:** József Haller

**Affiliations:** ^1^Department of Behavioural Neurobiology, Institute of Experimental Medicine, Hungarian Academy of Sciences, Budapest, Hungary; ^2^Institute of Behavioural Sciences and Law Enforcement, National University of Public Service, Budapest, Hungary

**Keywords:** violence, aggression, hypothalamus, humans, rodents

## Abstract

This review argues for a central role of the lateral hypothalamus in those deviant forms of aggression, which result from chronic glucocorticoid deficiency. Currently, this nucleus is considered a key region of the mechanisms that control predatory aggression. However, recent findings demonstrate that it is strongly activated by aggression in subjects with a chronically downregulated hypothalamus-pituitary-adrenocortical (HPA) axis; moreover, this activation is causally involved in the emergence of violent aggression. The review has two parts. In the first part, we review human findings demonstrating that under certain conditions, strong stressors downregulate the HPA-axis on the long run, and that the resulting glucocorticoid deficiency is associated with violent aggression including aggressive delinquency and aggression-related psychopathologies. The second part addresses neural mechanisms in animals. We show that the experimental downregulation of HPA-axis function elicits violent aggression in rodents, and the activation of the brain circuitry that originally subserves predatory aggression accompanies this change. The lateral hypothalamus is not only an integral part of this circuitry, but can elicit deviant and violent forms of aggression. Finally, we formulate a hypothesis on the pathway that connects unfavorable social conditions to violent aggression via the neural circuitry that includes the lateral hypothalamus.

## Introduction

Early studies by Hess (1928) attributed the hypothalamus a central role in aggression control, by showing that the electrical stimulation of particular hypothalamic sites rapidly induces biting attacks. This methodology contributed to aggression research by delimitating aggression-related hypothalamic regions, and by elucidating the major components of aggression-related neural networks (for reviews see Kruk, 1991; Siegel et al., 1999). Initially, hypothalamic regions involved in attack behavior were divided into two separate mechanisms. Mediobasal aspects of the hypothalamus (the mediobasal hypothalamus in cats, the hypothalamic attack area in rats, and the ventrolateral part of the ventromedial hypothalamus in mice) were shown to control intraspecific aggression. Albeit disparate early studies suggested that the electric stimulation of the lateral hypothalamus might also promote intraspecific aggression (Woodworth, 1971; Koolhaas, 1978), the effect remained somewhat elusive and occurred at rather large current intensities. Consequently, the consensus is that the lateral hypothalamus (in both cats and rats) controls predatory aggression (Smith et al., 1970; Kruk et al., 1979; Shaikh et al., 1987; Lin et al., 2011). In all the species studied so far (including humans) hypothalamic areas located medioventrally to the fornix were associated with intraspecific aggression, whereas hypothalamic areas located laterally to the fornix were shown to control interspecific (predatory) aggression (Haller, 2013).

**Figure 1:**
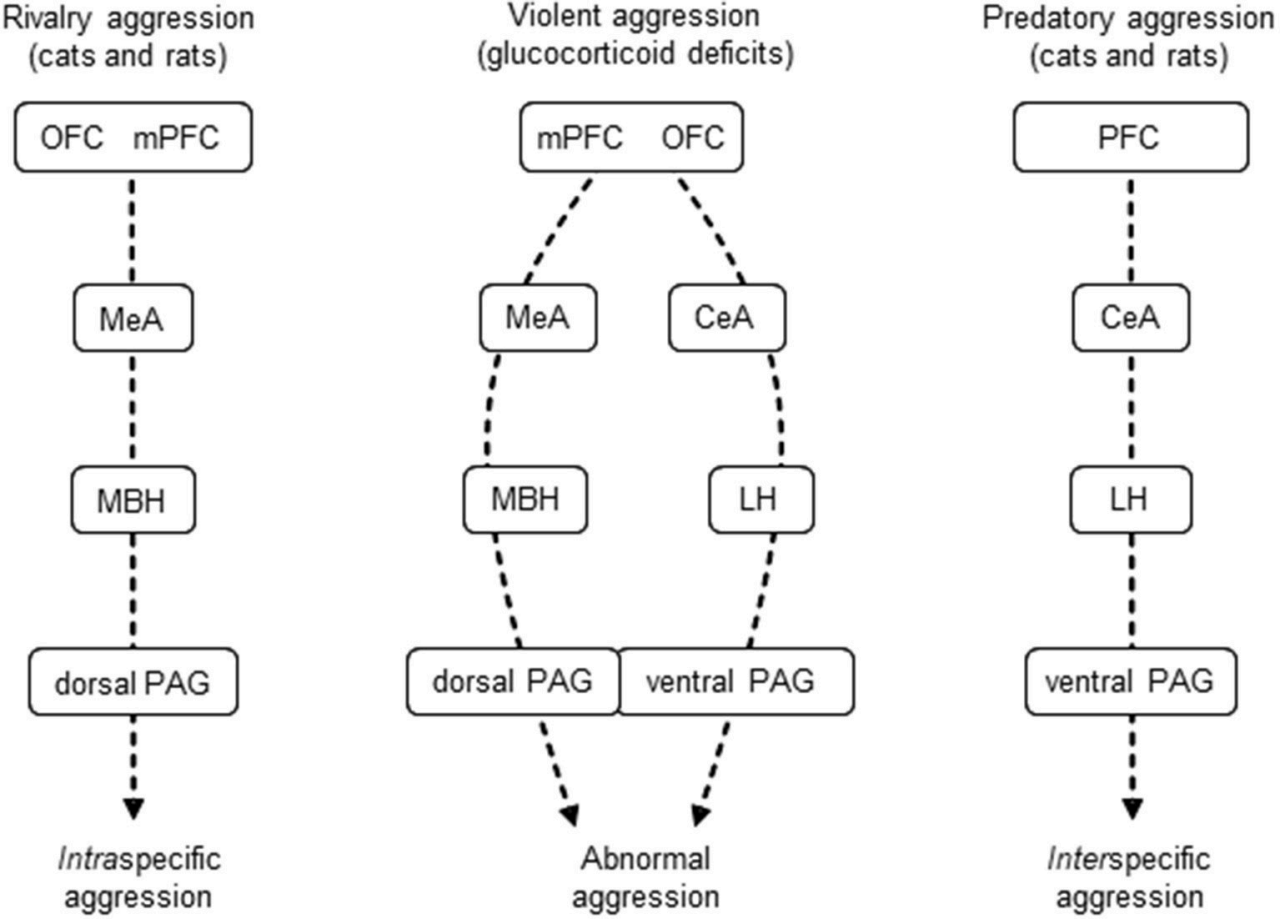
Mechanisms of glucocorticoid deficit-induced aggression are combinations of those subserving intraspecific and predatory aggressions. For explanations, see section Introduction. Dashed arrows indicate hypothetical information flow. CeA, central amygdala; LH, lateral hypothalamus; MBH, mediobasal hypothalamus (hypothalamic attack area); MeA, medial amygdala; mPFC, medial prefrontal cortex; OFC, orbitofrontal cortex; PAG, periaqueductal gray. Dashed arrows, hypothetical flow of information.

Our recent studies, however, indicated that the lateral hypothalamus is involved also in the control of intraspecific aggression, particularly in its violent forms. We found that the lateral hypothalamus was strongly activated during resident-intruder conflicts, but only if subjects were submitted to the glucocorticoid hypofunction model of abnormal aggression (Tulogdi et al., 2010, 2015). In this model of abnormal aggression, rats attack vulnerable body parts of opponents (head, throat band belly) without appropriately signaling attack intentions by social signals. We also showed that a subpopulation of prefrontal neurons specifically project to, and influence violent aggression controlled by the lateral hypothalamus (Biro et al., 2017, 2018). We recently proposed that the control mechanisms of violent forms of aggression are combinations of the mechanisms that subserve intraspecific (rivalry) and interspecific (predatory) aggressions (Haller, 2017; Figure 1).

The present review argues in favor of the role of the lateral hypothalamus in violent aggression, specifically in aggression associated with glucocorticoid deficits. The first part of the review is devoted to the role of glucocorticoid deficits in human aggression. The ultimate aim of this section is to clarify the translational value of the mechanisms discussed in the second part of the review. This latter section will in fact provide explanations for, and arguments supporting the mechanisms schematically represented in Figure 1. We suggest that glucocorticoid deficits are important contributors of deviant aggression in humans, and that animal findings with translational value suggest that the lateral hypothalamus has an important role in mediating the violence-related roles of glucocorticoid deficits.

## Glucocorticoid deficits and aggression in humans

Virkunnen reported in 1985 that cortisol secretion by habitually violent offenders with antisocial personality disorder was considerably lower than that of all three antisocial personality disordered subjects without habitual aggression, recidivist arsonists, and male clinic personnel. This report was published at a time when current thought associated aggression with increased rather than decreased stress responses (Posner and Conway, 1981; Public Health Report, 1983; Mason and Blankenship, 1987; Susman et al., 1988). Not surprisingly, reference to the now classical work by Virkkunen (1985) had a slow start. The first independent citation (a review) was published 4 years later (Zuckerman, 1989), and it required about a decade till the first confirmatory studies were published (Vanyukov et al., 1993; van Goozen et al., 1998; McBurnett et al., 2000; Dolan et al., 2001; Pajer et al., 2001; Kariyawasam et al., 2002). More than a decade elapsed until the first laboratory model of the condition was developed (Haller et al., 2001, 2004), albeit associations between low glucocorticoid levels and aggression were observed earlier (Poole and Brain, 1974).

Taken together, these findings suggested that certain types of human aggression and aggression-related psychopathologies are associated with downregulated hypothalamus- pituitary-adrenocortical (HPA) axis function, and that mimicking this condition in rodents leads to the development of abnormal forms of aggression (Haller, 2014). Several authors questioned the assumption that low glucocorticoid levels are associated with aggression problems (Klimes-Dougan et al., 2001; von der Pahlen, 2005; Marsman et al., 2008), and indeed, findings are often contradictory, especially in children diagnosed with various aggression-related psychopathologies (Table 1). Nevertheless, the findings presented in Table 1 clearly demonstrate that such psychopathologies are associated with downregulated HPA-axis function in certain study populations at least, which raises the question of how such conditions develop.

**Table 1:** Cross sectional studies in children: associations between aggression-related psychological conditions and cortisol plasma levels.

**Condition**		**Cortisol measurement**						**Association**	**References**
**Primary**	**Secondary**	**Single**	**Multiple**	**Diurnal**	**Total**	**Awakening**	**Stress response**		
CD		X						↑	van Bokhoven et al., 2005
DIS		X						→	Scerbo and Kolko, 1994
ADHD	ODD	X						↓	Kariyawasam et al., 2002
CD		X						↓	Vanyukov et al., 1993
CD	CU	X						↓	Loney et al., 2006
CD	ODD, AGG	X						↓	Oosterlaan et al., 2005
CD			X					↓	Pajer et al., 2001
CD			X					↓	Pajer et al., 2006
DIS	ANX		X				X	↓^**^	Schoorl et al., 2016
CD				X				→	Fairchild et al., 2008
UNR	AGG			X				→	Van den Bergh et al., 2008
UNR	AGG			X				↓	Oberle et al., 2017
UNR	EXT			X				↓	Martin et al., 2014
DIS					X			→	Kruesi et al., 1989
UNR	EXT				X			↓	Puetz et al., 2017
UNR	EXT					X		↑	Marsman et al., 2008
UNR	EXT					X		→	Klimes-Dougan et al., 2001
CD	CU					X		↓	von Polier et al., 2013
UNR	EXT					X		↓	Cicchetti and Rogosch, 2001
DIS	AGG					X		↓	Van de Wiel et al., 2004
UNR	EXT					X		↓	Puetz et al., 2016
DIS							X	↑^‡^	McBurnett et al., 2005
ADHD							X	↓^‡^	Pesonen et al., 2011
ADHD	CD						X	↓^*^	Northover et al., 2016
CD							X	↓^†^	Yang et al., 2007
DIS							X	↓	van Goozen et al., 1998
DIS							X	↓^‡^	van Goozen et al., 2000
ODD							X	↓^$^	Snoek et al., 2002
ADHD	CU						X	↓^€^	Stadler et al., 2011

***Psychiatric/psychological conditions**. ADHD, attention deficit hyperactivity disorder; AGG, aggression; ANX, anxiety; CD, conduct disorder; CU, callous emotional traits, DIS, disruptive behavior; EXT, externalizing behavior; ODD, oppositional-defiant disorder; UNR, unreferred. **Cortisol measurements**. X, type of measurement performed; single, condition associated with a single cortisol measurement; multiple, condition associated with averaged multiple cortisol measurements; diurnal, condition associated with altered diurnal secretion rhythm; total, 24 h urinary cortisol or equivalent; awakening, diurnal increase in cortisol measured in the morning; stress response, stress response independent from aggression. **Association with cortisol**. ↓, condition associated with downregulated HPA-axis function; → , condition not associated with changes in HPA-axis function; ↑, condition associated with upregulated HPA-axis function*;

* *association with CD traits*;

** *complex interaction with anxiety*;

† *anticipation of stress (e.g., public speaking)*;

‡ *psychological challenge*;

$ *pharmacological challenge*;

€ *psychological stress, association with CU traits*.

### Conditions leading to glucocorticoid deficits in humans

Yehuda et al. (1990) were the first to suggest that on the long-run stressors may decrease HPA-axis function. They observed that cortisol secretion was significantly lower in post-traumatic stress disorder (PTSD) patients than in controls; moreover, cortisol levels correlated with PTSD symptomatology. Importantly, a recent paper from the same lab suggested that glucocorticoid treatments can ameliorate the symptoms of PTSD, suggesting causal relationships between the hormonal response and psychiatric symptoms (Yehuda et al., 2015). Despite these early findings in PTSD, more than a decade later Gunnar and Vazquez (2001) still felt the need to encourage those who observed similar phenomena. They wrote “Lower than expected cortisol values should not necessarily be relegated to the file drawer because they contradict the central dogma that stress must be associated with elevations in cortisol” (Gunnar and Vazquez, 2001). Scientific opinion has changed considerably currently; an increasing number of stressors that lower HPA-axis activity have been reported since.

#### A brief account of the findings

Detrimental conditions acquired via the mother such as exposure to various illicit drugs, caffeine, nicotine, alcohol, as well as synthetic glucocorticoids (administered as medications) were all shown to inhibit HPA-axis function in adulthood (Kapoor et al., 2008; Vázquez et al., 2012; Zhang et al., 2014; Buckingham-Howes et al., 2016).

Deficient parental care also inhibits HPA-axis function on the long run (Van der Vegt et al., 2009; Koss et al., 2014; Martin et al., 2014; McLaughlin et al., 2015); moreover, this effect becomes irreversible if “treatment” by adoption comes too late e.g., after the age of 2–3 years (McLaughlin et al., 2015). Family tradition of aggressiveness (Saxbe et al., 2012; Arbel et al., 2016) as well as parental psychopathology (antisocial personality disorder, and post-traumatic stress disorder) also decrease HPA-axis function on the long run (Vanyukov et al., 1993; Cordero et al., 2017). Various forms of early maltreatment e.g., neglect, and all forms of abuse (emotional, physical, sexual abuse) as well as traumatic stressors have a similar effect (Weissbecker et al., 2006; Elzinga et al., 2008; Carpenter et al., 2009; Bosch et al., 2012; Lovallo et al., 2012; Doom et al., 2014; Trickett et al., 2014; Peckins et al., 2015; Puetz et al., 2016).

Extra-familial conditions, e.g., violent neighborhoods and community violence in general, bullying in schools, the proximity of pubs were all negatively associated with indices of HPA-axis function in the adulthood of subjects (*blunted stress responses*: Busso et al., 2017; *blunted cortisol responses*: Ouellet-Morin et al., 2011; Janusek et al., 2017; *steeper diurnal decline in cortisol levels during the day*: Theall et al., 2017; *low basal cortisol levels*: Vaillancourt et al., 2008).

Finally, adversities suffered in adulthood also decrease HPA-axis function on the long run (Carsia and McIlroy, 1998; Dayan et al., 2016; Pinto et al., 2016; Ewing-Cobbs et al., 2017). Such stressors include malnutrition, intimate partner violence, traumatic brain injury, and traumatic stress. HPA-axis function also showed chronic deficits in certain psychopathologies e.g., antisocial personality disorder, atypical depression, chronic fatigue syndrome, cocaine addiction, post-traumatic stress disorder, and schizophrenia (Buydens-Branchey et al., 1997; Yehuda, 1999; Strous et al., 2004; Bührsch et al., 2009; Flory et al., 2009; Nijhof et al., 2014; Berger et al., 2016; Juruena et al., 2017; Pruessner et al., 2017; Das et al., 2018). While the association was likely expected with certain disorders (e.g., antisocial personality and post-traumatic stress disorders), others may need some clarification. Depression overall is clearly associated with chronic stress; yet depressed patients who suffered strong stressful events in their early life showed decreased awakening cortisol in adulthood (Strous et al., 2004). Noteworthy, the awakening-induced increase in cortisol production is believed to reflect best the overall functional status of the HPA-axis as it is free of confounds from daily events (Kudielka and Wüst, 2010). Decreased HPA-axis function was also observed in atypical depression, a condition that may be associated with aggressiveness (Hasler et al., 2004; Juruena et al., 2017). Although stressors were earlier associated with the *development* of schizophrenia, it was recently shown that *the disorder per se* is associated with low cortisol plasma levels throughout the day–possibly as a response to early stress exposure (Strous et al., 2004; Berger et al., 2016; Pruessner et al., 2017; Das et al., 2018). Moreover, decreased cortisol levels were shown to contribute to aggression in psychosis. Finally, cocaine addiction *per se* was not associated with decreased plasma levels of cortisol, but aggressive addicts had a blunted cortisol response to meta-chlorophenylpiperazine challenge (Buydens-Branchey et al., 1997).

#### Overall evaluation of findings

HPA-axis deficits evidenced by the studies briefly reviewed above may take many forms, from decreased morning cortisol and blunted awakening response, trough decreased levels over the day to blunted cortisol responses to various types of challenges. Although the relative importance of such individual events is unclear at present, it should be noted that one and same stressful event elicited the full range of changes albeit in different studies. For example, early adversity decreased cortisol stress responses in some studies (Elzinga et al., 2008; Trickett et al., 2014), decreased basal levels or flattened the diurnal rhythm of cortisol secretion in others (Sánchez et al., 2005; Bosch et al., 2012; Peckins et al., 2015), and decreased morning cortisol in yet other studies (Weissbecker et al., 2006; Puetz et al., 2017). As such, effects may cover a variety of indices of HPA-axis deficits, even if one particular aspect was addressed in a particular study. Taken together, the findings demonstrate that various stressors suffered from prenatal to adult ages can inhibit the function of the HPA-axis on the long run.

Albeit the studies reviewed above were primarily endocrinological in their scope, some of them did investigate the behavior of subjects. In all such studies, low HPA-axis function was associated with indices of aggression (*bullying*: Ouellet-Morin et al., 2011; *cocaine addicts with low stress responses*: Buydens-Branchey et al., 1997; *early life stress*: Puetz et al., 2016; *poor parental monitoring*: Martin et al., 2014; *prenatal drug exposure*: Buckingham-Howes et al., 2016; *prenatal synthetic glucocorticoids*: Kapoor et al., 2008; *schizophrenia*: Strous et al., 2004; Das et al., 2018). In these studies, aggression was conceptualized as problem behavior, social and behavioral problems, externalizing behavior, high scores on aggression inventories, the display of aggression-related psychopathologies, and aggression histories. Particular behavioral descriptions were usually not provided. Thus, long-term decreases in HPA-axis function was associated with various indices of aggressiveness.

#### Comparison of HPA-axis down- and upregulating factors

Stressful events that led to a downregulated HPA-axis on the long run (see above) are surprisingly similar to those, which augmented HPA-axis function in other studies (see the reviews by Kuhlman et al., 2017; Raymond et al., 2017). This leads to the surprising conclusion that one and same stressor may down—or upregulate the stress system on the long run, depending on an unknown array of circumstances. While such circumstances cannot be identified at present, the available data provide some hints toward the solution of the contrasting findings.

The first aspect to be considered is related to the temporal evolution of events. It has been repeatedly shown that stressors that upregulate the HPA-axis may ultimately lead to its downregulation. For instance, caffeine, nicotine or alcohol consumed by the mother enhanced cortisol secretion in both the mother and the infant; blunted stress responses were observed only when subjects reached adulthood (Zhang et al., 2014). In the study by Bosch et al. (2012), pre- and post-natal stressors also elicited a cortisol response acutely. Subsequently, the adversity was not associated with cortisol outcomes up to the age of 5, the upregulation of the HPA-axis was observed up to the age of 11, whereas a few years thereafter low cortisol levels were observed in subjects. Although not detailed to a similar degree, the trajectory of HPA-axis changes followed the same pattern in a number of cases. Acute and (sub)chronic stress responses were followed over time by a downregulation of the HPA-axis (Gunnar and Vazquez, 2001; Fries et al., 2005; Sánchez et al., 2005; Doom et al., 2014; Zhang, 2017). These findings show that the glucocorticoid deficit develops over time, and subjects frequently reach this phase after a period of HPA-axis upregulation. It also occurs that the process may take years.

Another factor that should be taken into account is the variability in HPA-axis responses. Peckins et al. (2015) categorized their subjects with respect to stress responses rather than calculating overall averages. They identified three cortisol profiles emerging after stress exposure (blunted, moderate, and elevated stress responses), and found that maltreated youth were more likely to show a blunted cortisol profile on the long run, but some of them fell into the other two categories. Van der Vegt et al. (2009) found that the magnitude of HPA-axis dysregulation depended largely on the severity of early maltreatment. Individual variability in HPA-axis responses to stress was observed in other studies as well (Weissbecker et al., 2006; Flory et al., 2009). Thus, the direction of HPA-axis changes (up- or down-regulation) and the magnitude of the effect strongly depend on the individual features of subjects, and/or to life events subsequent to the initial stressor. Naturally, other factors e.g., genetic susceptibility, and the type of stress are also important (see Daskalakis et al., 2013 for a review).

#### Conclusions

The long-term decrease in HPA-axis function that develops in response to strong stressors may be best conceptualized as an “allostatic crash,” a term coined by Van Houdenhove et al. (2009). This concept suggests that severe stressors, when associated with low individual resilience lead to an allostatic load that overpasses the adaptive capacities of the HPA-axis. This—metaphorically saying—results in the “fatigue” or “burnout” of the system. Such a “burnout” may be facilitated by the transient phase of upregulation (see above) or by subsequent stressors. Collectively, the excessive allostatic load leads to a failure of the adrenals to produce adequate quantities of glucocorticoids under basal conditions, and/or to failures of the system to adequately respond to acute stressors.

The likely factors that differentiate conditions that lead to the chronic upregulation or the chronic downregulation of the HPA-axis are the severity and duration of the triggering event (the initial stressor), individual susceptibility to stress effects, and life events that follow the initial stressor. Their relationship may place the HPA-axis on opposing trajectories and may lead either to the up- or to the downregulation of the HPA-axis.

### Glucocorticoid deficits and human aggression

The study by Virkkunen (1985) implicitly suggests that glucocorticoid deficiency is associated with severe aggression; moreover, superimposed deficits e.g., antisocial personality disorder and habitual offending. Some recent findings also imply that HPA-axis hypoactivity characterizes a particularly severe subgroup of subjects who show both chronic antisocial behavior and callous-unemotional traits (Hawes et al., 2009). Other evidence, however, suggests that glucocorticoid deficits can be associated with less severe forms of aggression.

#### A brief account on findings

By a thorough Medline search using various keywords, we identified 76 studies on the interaction between HPA-axis function and aggressiveness. We summarized findings in Tables 1–**4**. Individual tables refer to studies in particular subjects; Table 1 for instance shows cross-sectional studies performed in children who showed various aggression-related conditions. Findings were arranged according to the method of cortisol measurements to visualize possible confound resulting from this variable. As argued elsewhere (Haller et al., 1998, 2008; Haller, 2014), types of measurement are not equivalent. Single measurements appear to be the least reliable due to accidental variations in plasma cortisol levels. Averaged multiple measurements eliminate such confounds, whereas awakening cortisol as well as indices of total cortisol production (e.g., 24 h urinary cortisol) can be considered general indices of HPA-axis function. Diurnal variations provide detailed information, which may reveal alterations specific to particular phases of the day. Finally, the magnitude of the stress response may vary independently from basal levels, by this providing a yet different measure of HPA-axis function.

Out of the 29 cross-sectional studies performed in children, 21 (72.4%) revealed a negative association between HPA-axis function and conditions associated with aggressiveness; 5 (17.3%) found no association, whereas the opposite relationship was reported in the remaining 3 studies (10.3%) (Table 1). The method of glucocorticoid measurement does not seem to have a role in discrepancies, because all the 6 ways of HPA-axis evaluation was found in all the three categories of interactions. Out of the primary conditions (diagnoses) investigated, attention deficit hyperactivity disorder (ADHD) showed the negative association consistently, whereas findings in disruptive behavior were the least consistent. Findings in unreferred subjects evaluated for externalizing behavior were rather inconsistent as well. Out of the secondary conditions, callous-unemotional traits showed the negative association consistently whereas findings in externalizing behavior were the least consistent. Studies lacking a secondary moderating factor also provided inconsistent findings.

Taken together, the findings summarized in Table 1 show that the association between aggression-related conditions and HPA-axis function are conflicting in cross-sectional studies performed in children. Albeit the overwhelming majority of studies reported a negative association, a significant proportion of studies are at variance with this conclusion. No contradictions were found with certain conditions e.g., ADHD and callous unemotional traits, but the number of relevant studies is too small to draw definite conclusions regarding these conditions.

In sharp contrast to cross-sectional studies, longitudinal ones performed in children appear highly consistent (Table 2). In the majority of these studies, unreferred subjects were studied at time-point 1, when there was no interaction between cortisol levels and behavior. Note that no problem behavior was identified at this time-point. Low levels of cortisol at time-point 1, however, predicted the development of problem behaviors at the 2nd time-point i.e., more than 2 years later (up to 6 years in some studies). There were only two exceptions, but neither produced conflicting findings. In one study, childhood internalizing behavior predicted downregulated HPA-axis function in adolescence; no similar association was found for externalizing behavior (Ruttle et al., 2011). In the second study, the negative association was observed at the first time-point already, and this was maintained over time (Salis et al., 2016). Noteworthy, however, the subjects of this study were adolescents at the first time-point. One can hypothesize that the prediction of problem behavior by low cortisol was valid for these subjects as well, but they were over the critical age. Again, the method of cortisol measurement did not affect the relationship between cortisol and behavior.

**Table 2:** Longitudinal studies in children: associations between cortisol plasma levels and long-term changes in aggression-related conditions.

**Condition**		**Cortisol measurement**						**Association**		**References**
**Concurrent**	**Longitudinal**	**Single**	**Multiple**	**Diurnal rhythm**	**Daily production**	**Awakening response**	**Stress response**	**Concurrent**	**Longitudinal**	
UNR	DIS	X						→	↓	Sondeijker et al., 2008
UNR	DIS		X					→	↓	McBurnett et al., 2000
UNR	DIS		X					→	↓	Alink et al., 2012
UNR	EXT		X					→	↓	Shirtcliff and Essex, 2008
UNR	EXT		X					→	↓	Shirtcliff et al., 2005
UNR	AGG		X					→	↓	Shoal et al., 2003
INT	INT		X					→	↓^*^	Ruttle et al., 2011
UNR	AGG			X				→	↓	Salis et al., 2016
AGG	AGG					X		↓	↓^**^	Platje et al., 2013

***Temporal relationships**. Concurrent, first time point of the study; longitudinal, subsequent time-points of the study (usually years after “current”). **Psychological conditions**. AGG, aggression; DIS, disruptive behavior; EXT, externalizing behavior; INT, internalizing behavior; UNR, unreferred. **Cortisol measurements**. X, type of measurement performed; single, condition associated with a single cortisol measurement; multiple, condition associated with averaged multiple cortisol measurements; diurnal, condition associated with altered diurnal secretion rhythm; total, 24 h urinary cortisol or equivalent; awakening, diurnal increase in cortisol measured in the morning; stress response, stress response independent from aggression. **Association with cortisol**. → , condition not associated with changes in HPA-axis function concurrently (i.e., during the first time-point of the study); ↓, the condition associated with downregulated HPA-axis function over time (cortisol levels at the first time point were compared with psychiatric conditions observed in subsequent time-points)*;

* *increase in internalizing behavior predicted the decrease in cortisol plasma levels over time*;

** *study subjects were adolescents (16–19 years old)*.

Out of the 19 studies performed in adolescents and adults, low HPA-axis functioning was associated with various indices of aggressiveness in 18 studies (Table 3). The only exception was a study performed in antisocial personality-disordered subjects, where psychopathic traits did not correlate with the diurnal patterns of cortisol secretion (Loomans et al., 2016). The lack of interaction cannot be attributed to the way of cortisol measurement, as two other studies employed the same approach; moreover, one of these found associations with psychopathic traits (Vaillancourt and Sunderani, 2011; Das et al., 2018). This discrepancy is difficult to explain. However, the rest of the studies are remarkably consistent as it regards the association between aggressiveness and cortisol, despite the large variation in subject types (from unreferred through psychotic and drug addiction to antisocial personality disorder) and the various ways of HPA-axis evaluations.

**Table 3:** Studies in adolescents and adults: associations between plasma cortisol and aggression-related psychiatric conditions.

**Condition**		**Cortisol measurement**						**Interaction**	**References**
**Primary**	**Secondary**	**Single**	**Multiple**	**Diurnal rhythm**	Daily production^*^	**Awakening**	**Stress responses**		
UNR	AGG	X						↓	Yu and Shi, 2009
ALC	VIOL	X						↓	Bergman and Brismar, 1994
UNR	PP	X						↓^*^	Glenn et al., 2011
SCH	AGG	X						↓	Strous et al., 2004
UNR	CU		X					↓^**^	Fanti and Kimonis, 2017
UNR	AGG		X					↓	Victoroff et al., 2011
UNR	PP1			X				↓^†^	Vaillancourt and Sunderani, 2011
PSYCH	VIOL			X		X		↓	Das et al., 2018
APD	PP			X				→	Loomans et al., 2016
UNR	AGG				X			↓^*^	Grotzinger et al., 2018
UNR	AGG						X	↓^‡^	Böhnke et al., 2010
UNR	AGG						X	↓^$^	Gordis et al., 2006
HERadd (ABST)	AGG						X	↓^‡^	Gerra et al., 2004
HERadd (METH)	AGG						X	↓^‡^	Gerra et al., 2001
APD	-						X	↓^€^	Almeida et al., 2010
UNR	EXT						X	↓^*^	Portnoy et al., 2015
UNR	PP						X	↓	O'Leary et al., 2007
COCadd	AGG						X	↓^€^	Buydens-Branchey et al., 1997
PDE	AGG, EXT						X	↓	Buckingham-Howes et al., 2016

***Psychiatric/psychological conditions**. ABST, abstinent at the time of the study; AGG, aggression; ALC, alcoholic; APD, antisocial personality disorder; CU, callous emotional traits, EXT, externalizing behavior; HERadd, heroin addiction; METH, on methadone at the time of the study; PDE, prenatal drug exposure; PP, psychopathic traits; PP1, psychopathy type 1; PSYCH, psychosis; SCH, schizophrenia; UNR, unreferred; VIOL, violence. **Cortisol measurements**. X, type of measurement performed; single, condition associated with a single cortisol measurement; multiple, condition associated with averaged multiple cortisol measurements; diurnal, condition associated with altered diurnal secretion rhythm; total, 24 h urinary cortisol or equivalent; awakening, diurnal increase in cortisol measured in the morning; stress response, stress response independent from aggression. **Association with cortisol**. ↓, condition associated with downregulated HPA-axis function; → , condition not associated with changes in HPA-axis function; ↑, condition associated with upregulated HPA-axis function*;

* *association with testosterone/cortisol ratio*;

** *association with externalizing and internalizing behaviors*;

† *females only*;

‡ *experimental provocation unrelated to aggressiveness*;

$ *interaction dependent on alpha amylase activity*;

€ *pharmacological challenge*.

Finally, findings obtained in delinquents also appear rather consistent (Table 4). Out of the 18 studies, delinquents had downregulated HPA-axes in 15 (83%). Interestingly, the association did not depend on the type of delinquency, as low HPA-axis function was observed in both violent and nonviolent offender populations. In two studies, no association was observed (Feilhauer et al., 2013; Gostisha et al., 2014). However, the impact of antisocial and psychopathic traits was investigated in these studies. If HPA-axis deficits were associated with delinquency *per se* (as the rest of the studies suggest), the lack of an additional impact by such traits becomes explainable. The only finding that was discrepant indeed reported that violent delinquents show enhanced stress responses (Soderstrom et al., 2004). The discrepancy cannot be due to the way of HPA-axis evaluation as four other studies found decreased stress responses in delinquent populations; these were done either in violent or non-violent offenders (Moss et al., 1995; Popma et al., 2006; Couture et al., 2008; Johnson et al., 2015).

**Table 4:** Studies in delinquent populations: associations between plasma cortisol and delinquency.

**Condition**	**Cortisol measurement**						**Interaction**	**References**
**Delinquent type**	**Single**	**Multiple**	**Diurnal rhythm**	Daily production^*^	**Awakening response**	**Stress responses**		
M/nV	X						↓^*^	Popma et al., 2007b
M/nV	X						↓^**^	Poustka et al., 2010
M/nV	X						↓	Dolan et al., 2001
M/nV	X						↓^†^	Horn et al., 2014
M/nV	X					X	↓^‡^	Couture et al., 2018
PP		X					↓^*^	Dabbs et al., 1991
PP		X					→	Feilhauer et al., 2013
ANTS			X				→^₳^	Gostisha et al., 2014
M/nV			X				↓	Popma et al., 2007a
VIOL			X				↓	Brewer-Smyth et al., 2004
PP			X				↓	Cima et al., 2008
VIOL				X			↓	Virkkunen, 1985
VIOL					X		↓^$^	Holi et al., 2006
M/nV						X	↓^€^	Popma et al., 2006
VIOL						X	↓^€^	Moss et al., 1995
VIOL						X	↑^€^	Soderstrom et al. (2004)
M/nV						X	↓^¥^	Johnson et al., 2015
M/nV						X	↓^‡^	Couture et al., 2008

***Delinquent type**. ANTS, delinquents with antisocial traits; M / nV, mixed delinquent population, committing mostly non-violent crime; PP, delinquents with psychopathic traits; VIOL, violent crime. **Cortisol measurements**. X, type of measurement performed; single, condition associated with a single cortisol measurement; multiple, condition associated with averaged multiple cortisol measurements; diurnal, condition associated with altered diurnal secretion rhythm; total, 24 h urinary cortisol or equivalent; awakening, diurnal increase in cortisol measured in the morning; stress response, stress response independent from aggression. **Association with cortisol**. ↓, delinquency associated with downregulated HPA-axis function; ↑, delinquency associated with upregulated HPA-axis function*;

* *interaction with testosterone/cortisol ratio*;

** *males only*;

† *interactions mediated by personality and substance use disorders*;

‡ *interaction mediated by risk taking*;

₳ *, complex interactions with stress exposure*;

$ *interaction with psychopathic features*;

€ *anticipation of stress (e.g., public speaking)*;

¥ *interaction with the number of incarcerations*.

Taken together, conditions associated with aggressiveness or violence show a more reliable association with downregulated HPA-axis than generally believed (Klimes-Dougan et al., 2001; von der Pahlen, 2005; Marsman et al., 2008). The findings briefly reviewed above reveal that the negative association is neither restricted to extreme violence nor to callus-unemotional traits as previously suggested (Virkkunen, 1985; Hawes et al., 2009). Moreover, it may be valid for delinquents in general, irrespective to the violent nature of offenses committed. At the same time, however, there are a series of discrepant findings as well. Although these represent a rather small share of the studies, there are no obvious reasons to neglect them.

#### Discrepant findings—possible explanations

One possible explanation to the discrepant findings may reside in the putative long-term association of glucocorticoid deficits and aggressiveness. Such findings were summarized in Table 2. One can hypothesize that the negative association between glucocorticoid production and aggressiveness may not be obvious in some cases because the behavioral change is subsequent to the decrease in glucocorticoid production, and the temporal difference between the two can be measured in years. This may at least partly explain discrepant findings in children. However, discrepant findings were reported at older ages too. Another explanation may reside in the type of aggression performed by subjects, which is considered rather rarely.

Aggression is often divided into two types: instrumental (proactive) and emotional (reactive). The former is non-impulsive and gain oriented, whereas the latter is a response to threat or provocation, is impulsive, and is not performed for gain (Feshbach, 1971; Blair, 2001; Kempes et al., 2005; Raine et al., 2006; van Honk et al., 2010). The two forms of aggression appear to have a differential association with cortisol. Instrumental (proactive) aggression appears associated with blunted, whereas emotional (reactive) aggression seems to be associated with upregulated HPA-axis function (McBurnett et al., 2003, 2005; Kempes et al., 2005; van Bokhoven et al., 2005; Lopez-Duran et al., 2009; O'Neal et al., 2010; Poustka et al., 2010; van Honk et al., 2010; Geniole et al., 2011; Stoppelbein et al., 2014). Noteworthy, a similar dichotomy was found in animal models (Haller, 2016).

Evaluating the relationship between aggression and aggression-induced changes in cortisol production, as well as the relationship between reactive aggression and enhanced glucocorticoid production is outside the scope of this review, which aims at evaluating the role of the lateral hypothalamus in aggression performed under conditions of downregulated HPA-axis. However, studies where reactive and proactive forms of aggression were directly compared as it regards their glucocorticoid background strongly suggest that the two types of aggression substantially differ in this respect, and may explain discrepancies in the studies reviewed above.

### Overall evaluation of human findings

The studies briefly reviewed above show that on the long run, a large number of stressors elicits HPA-axis deficits in humans. These stressors coincide largely with those, which on the long run may also elicit a chronic enhancement of HPA-axis function. There are no explanations for such contrasting long-term trajectories of HPA-axis responses to stress, but disparate findings suggest that the severity of the “initial” stressor, as well as subsequent life events, together with individual vulnerabilities may have an important role. The long-term decrease in HPA-axis function may result from an “allostatic crash” i.e., from an allostatic load that overpasses the adaptive capacities of the HPA-axis. HPA-axis deficits develop slowly over time, and subjects often go through a phase of HPA-axis hyperfunction before the “final” glucocorticoid deficit develops, albeit this assumption requires further evidence.

Although initially suspected to be related to extreme aggression, a series of studies suggest that glucocorticoid deficiency is associated with a wide variety of aggressive behaviors. Overall, the findings suggest that the “prime suspects” are proactive (instrumental) forms of aggression. Psychopathic e.g., callous-unemotional traits strengthen the association. Glucocorticoid deficiency was repeatedly associated violent crime as well.

The available findings suggest that glucocorticoid deficiency precedes behavioral changes. The vast amount of evidence on the association of glucocorticoid deficiency with aggression as well as the precedence of hormonal over behavioral changes suggest a causal relationship. However, this assumption is based on indirect evidence only, and the nature of the phenomenon precludes the obtaining of direct evidence in humans. The issue requires animal experimentation.

## The role of the lateral hypothalamus

**Figure 2:**
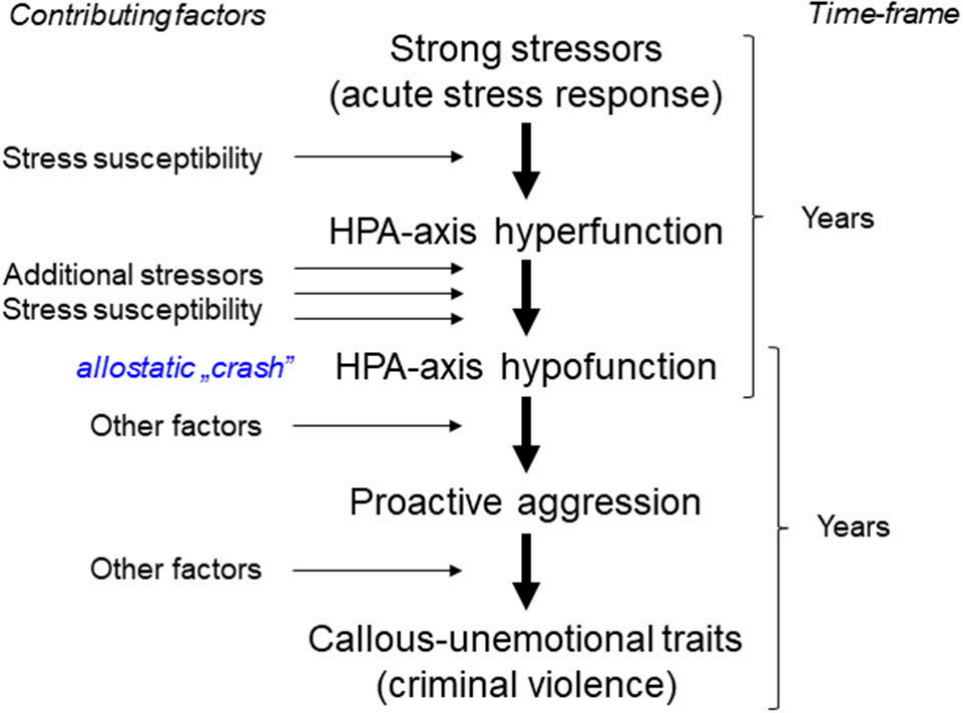
Hypothetical pathway from strong stressors to violent aggression through HPA-axis hypofunction. The latter is attributed to an “allostatic crash” (see section Glucocorticoid Deficits and Aggression in Humans). “Other factors” were included to indicate that hormonal conditions are likely embedded in a wider array of biological, psychological, and social factors. The time frame is based on a number of longitudinal studies performed in late childhood—early adolescence.

Findings in humans were tentatively summarized in Figure 2. In addition to factors that were based on the findings reviewed above, we added “other factors” to indicate that hormonal conditions are likely embedded in a wider array of biological, psychological, and social factors (Dodge et al., 1990; Loeber and Hay, 1997; van Honk et al., 2010).

Despite the clear involvement of the hypothalamus in the control of aggression, little effort was invested in understanding its role in humans. The available studies either focus on the “triangle of Sano” (a hypothalamic region, the destruction of which strongly inhibits aggressive behavior; Sano et al., 1970) or the technologies employed were unable to differentiate hypothalamic nuclei, and consequently considered it as a unit (Rosa et al., 2012; Van den Stock et al., 2015; Mutic et al., 2017). As the triangle of Sano includes parts of the lateral hypothalamus, but is not restricted to it, and the term “hypothalamus” is not sufficiently precise for the purposes of this study, we have to turn to animal models.

### The concept of abnormal aggression in animals

Over the last decades, rodent abnormal aggression models have increasingly been used to mimic symptoms of aggression-related psychopathologies. Such models (1) mimic in rodents etiological factors of human aggression problems; (2) study the resulting aggressiveness by focusing on the form of aggression and its behavioral contexts, and (3) strive at investigating aggression-independent features (e.g., autonomic responses, anxiety, and social behaviors beyond aggressiveness) which are important concomitants of aggression-related psychopathologies (for reviews see Haller et al., 2004, 2014; Miczek et al., 2013).

The overall conclusion deriving from such studies is that the etiological factors of human aggression problems result in abnormal forms of aggression in rodents. Human etiological factors modeled so far to study their effects on animal aggression include but are not restricted to alcohol, early social isolation, frustration by the omission of scheduled reward, glucocorticoid deficiency, peripubertal social or physical stressors, repeated maternal separation, repeated treatment with cocaine, amphetamine, or anabolic steroids during adolescence, and selection for extremes in aggression and anxiety to mimic the genetic component of human aggression (Benus et al., 1991; Miczek et al., 1992; Melloni and Ferris, 1996; Delville et al., 1998; Haller et al., 2001; de Almeida and Miczek, 2002; Ricci et al., 2005; Veenema et al., 2006; Tóth et al., 2008; Gobrogge et al., 2009; Neumann et al., 2010; Márquez et al., 2013). All these etiological factors elicit abnormal manifestations of aggression in rodents, which are perceived as models for the symptoms of aggression-related psychopathologies. As an example, the glucocorticoid deficiency model changed the behavior and physiology of subjects in the following ways. (1) Attack targeting shifted from less vulnerable body parts (back and flanks) to more vulnerable ones (head, throat, belly, and occasionally paws and testicles); (2) The propensity of signaling attack intentions by social signals (threats) decreased; (3) Attacks were performed under conditions of low autonomic arousal; (4) The social behavior of subjects showed important deficits (beyond those related to aggression). This array of features mimics important aspects of human proactive (antisocial) aggressiveness (Haller et al., 2004; Haller, 2017).

### Aggression models associated with glucocorticoid deficits

The glucocorticoid deficit model of abnormal aggression was purposefully developed to mimic the human condition discussed in this review (see above). In addition to this model, there are several other abnormal aggression models, where the induction of glucocorticoid deficits was not an aim, but deficient HPA-axis function was observed later on. This was observed in the early subjugation model of aggression (Ferris, 2003), in mouse lines selected for aggressiveness, particularly the short attack latency mice (Veenema et al., 2004; Natarajan and Caramaschi, 2010), and rats selected for extremes in anxiety (Neumann et al., 2010). In the pubertal stress model, corticosterone secretion was not decreased overall, but was decreased relative to testosterone secretion (Márquez et al., 2013).

Maternal aggression is also associated with low glucocorticoid stress responses (Neumann, 2001; Neumann et al., 2001); moreover, acute stressors suffered before the aggressive encounter and increased stress responsiveness (as an individual feature) decrease maternal aggression (Gammie et al., 2005; Gammie and Stevenson, 2006). Albeit maternal aggression cannot be considered abnormal, it shares several features with the glucocorticoid deficit model of abnormal aggression. When protecting their pups, dams attack vulnerable body part of their opponents, and fail to signal their attack intentions by social signals (Parmigiani et al., 1988). Finally, predatory aggression is also performed under low arousal, and consist of lethal attacks on vulnerable body parts (Siegel et al., 1999).

The studies briefly reviewed above suggest that in certain abnormal aggression models as well as in two models of natural aggressiveness (maternal and predatory aggressions) violent forms of aggression are associated with decreased HPA-axis function.

### Violent aggression and the lateral hypothalamus

Where the lateral hypothalamus was investigated, glucocorticoid deficit-associated violent aggression was also associated with a marked activation of this hypothalamic region (*glucocortioid deficit model*: Tulogdi et al., 2010; *mouse lines selected for aggressiveness*: Haller et al., 2006; *rats selected for extremes in anxiety*: Beiderbeck et al., 2012; *predatory aggression*: Tulogdi et al., 2015; *maternal aggression*: Hasen and Gammie, 2006). In these models, the mediobasal hypothalamus was also activated, with the exception of the predatory aggression model. A detailed analysis of activation patterns observed in aggression models associated with glucocorticoid deficit and those associated with normal or enhanced glucocorticoid responses revealed three different brain activation patterns (Haller, 2014, 2017). The overall conclusion of these studies was that: (1) regularly performed resident-intruder test activate the medial amygdala-mediobasal hypothalamus-dorsal periaqueductal gray pathway. (2) In abnormal aggression models associated with *increased* glucocorticoid stress responses, the same pathway was activated, but the medial amygdala and mediobasal hypothalamus together with certain areas of the prefrontal cortex and the basolateral amygdala showed increased activations. (3) Abnormal aggression models associated with *decreased* glucocorticoid stress responses activate the same pathway but in addition they also activate the central amygdala-lateral hypothalamus-ventral periaqueductal gray pathway. Finally (4) predatory aggression activates exclusively the central amygdala-lateral hypothalamus-ventral periaqueductal gray pathway (Figure 1).

Naturally, correlations between behavioral, endocrine and brain activation patterns are indicative of, but not proofs of causal relationships. Recent studies, however, do suggest such causal relationships. These studies were prompted by the finding that hypothalamic areas involved in aggression control receive direct inputs from the prefrontal cortex, a brain area tightly involved in aggression control (Sesack et al., 1989; Siegel et al., 2007; Toth et al., 2010). Blair (2001) attributed a large role of such direct prefrontal cortex–hypothalamus connections in his model of human reactive aggression, albeit subsequent studies by the same author suggest that the prefrontal cortex–hypothalamus link is not direct but is mediated by the amygdala (Blair, 2012, 2016). Recent rodent findings, however, appear to re-establish the notion that the prefrontal cortex controls aggression by direct effects on the hypothalamus, a mechanism that may complements the more established prefrontal cortex–amygdala–hypothalamus pathway (Biro et al., 2018). We established in a recent study that the hypothalamus is directly (mono-synaptically) innervated by the prefrontal cortex; moreover, the mediobasal and lateral hypothalamus received inputs from dominantly distinct prefrontal neuron populations. These neurons were located in the prelimbic and infralimbic region of the medial prefrontal cortex. Prefrontal projecting neurons were dispersed in the layers III–V of the infralimbic and prelimbic cortices, and their dense axon terminals at hypothalamic sites exclusively contained vesicular glutamate transporter 1, i.e., these neurons were glutamatergic. The optogenetic stimulation of mPFC terminals in the MBH distinctively increased bite counts in resident/intruder conflicts, whereas stimulation of similar terminals in LH specifically resulted in violent bites. These were aimed at vulnerable targets of opponents (head, throat and belly, occasionally paws), and/or were not preceded by social signals (threats). No other behavior was affected, suggesting that dedicated prefrontal neurons control particular aspects of aggressiveness via the hypothalamus in a highly selective manner. Besides shedding light on the way in which the prefrontal cortex controls aggressive behavior (e.g., by highly dedicated subcortical efferents), these findings are to our knowledge the first proof of a causal involvement of the lateral hypothalamus in intraspecific aggression. These findings are in agreement with the role of this brain area in violent aggression, which was assumed earlier based on brain activation patterns, but surprisingly show that subpopulations of prefrontal neurons may increase aggressiveness; moreover, may make it more violent. In principle, this finding is at variance with the prefrontal deficit theory of aggression (Blair, 2001; Siegel et al., 2007). The role of the prefrontal cortex in aggression control is outside the scope of this review, therefore this aspect of the findings will not be discussed here in detail. We showed earlier, however, that aggressiveness is associated with both chronic prefrontal deficits and increased acute aggression-induced prefrontal activation in abnormal aggression models (Biro et al., 2017), and repeatedly argued elsewhere, that the involvement of the prefrontal cortex in aggression control should be revised (Haller, 2014, 2017). Albeit chronic prefrontal deficits increase the likelihood and severity of aggressive behavior, this brain area is activated by aggressive behavior acutely; moreover, activation is stronger in models of abnormal aggression and in subjects with aggression-related psychopathologies. Based on these findings, one can hypothesize that HPA-axis hypofunction affects the aggression circuitry at least partly at the level of the prefrontal cortex.

## The impact of glucocorticoid deficits on aggression: putative mechanisms

The basic question raised by the findings reviewed above is how the mechanisms controlling aggression are shifted from the ones that control rivalry aggression to the ones that control predatory aggression under normal circumstances. Low HPA-axis activity after allostatic overload or experimental manipulations is not likely to cause behavioral maladaptation by itself. Glucocorticoids affect the properties of neurons rather than elicit membrane depolarizations or activate brain circuitries. Consequently, the basic question is how the absence of well-timed, adaptive adrenocortical response in the face of a social challenge or emergency alters the properties of aggression-controlling networks.

Some time ago we speculated that the dynamics of the endocrine responses accompanying social challenges and aggression,—most notably the dynamics of the HPA-axis—, would rapidly affect the way animals cope with actual and future conflicts. We also suggested that studying the underlying mechanisms at the level of the hypothalamus and its interactions with frontal systems could possibly contribute to our understanding of inadequate, maladaptive conflict behavior in humans (Kruk et al., 1998). We also suggested that the hypothalamus serves as a crucial link between frontal areas involved in memory, the appraisal of the environments the recognition of conspecifics and the execution of specific aggressive responses. Such frontal areas, as we suggested at that time could either facilitate or inhibit hypothalamic aggression in rats (Kruk et al., 1998). Since then animal studies have provided strong support for these ideas reviewed e.g., in Kruk (2014). The dynamics of the adrenocortical response facilitates hypothalamic,- as well as territorial aggression within the time frame of one single conflict (Kruk et al., 2004; Mikics et al., 2004) by an initially non-genomic rapid mechanism. In the absence of such a response inadequate conflict handling takes over. Rapid adrenocortical signaling via the mineralocorticoid receptor also enables the first emergence of an adaptive aggressive response in a novel environment (Kruk et al., 2013). More recent studies clearly suggested the involvement of structures “up-stream” from the hypothalamus, such as the amygdala and the frontal cortex, in such behavioral effects of the adrenocortical response (Biro et al., 2017, 2018; Mikics et al., 2018).

These experimental studies suggest that the precise timing of the adrenocortical response with respect to the timing of a social challenge determines the adaptive nature of the aggressive response. Therefore, it stands to reason to predict that the absence of a well-timed adrenocortical response as a consequence of repeated severe stressors and the ensuing allostatic crash, could also be a major factor in inadequate conflict handling in humans. Recent results from animal experiments summarized here, suggest that the behavioral changes accompanying an allostatic crash in humans could be due to a reorganization of hypothalamic connections with “upstream,” modulating circuits, resulting in a change from affective social conflict handling toward a predatory like responding in conflicts.

Albeit acute deficits in the regulatory roles of glucocorticoids may have effects *per se* via the so-called non-genomic effects (Haller et al., 2008), the consequences of HPA-axis downregulation for aggression appear to develop slowly. This renders slow glucocorticoid actions better candidates for mediating the behavioral effects of glucocorticoid deficits, even if such slow alterations interact with rapid effects (Joëls et al., 2013).

A typical example of slow glucocorticoid effects are the ones that are mediated by epigenetic mechanisms. For example, early adversity was shown to durably alter the epigenetic regulation of glucocorticoid signaling genes and to induce DNA methylation in various brain areas (Lutz and Turecki, 2014; Tyrka et al., 2016). Importantly, glucocorticoids control gene expression in the prefrontal cortex, the brain site that shapes behavioral strategies during social conflict (Costin et al., 2013; Biro et al., 2018). The assumption that epigenetic changes in the prefrontal cortex elicit abnormal aggression is supported by recent findings showing that such behaviors largely depend on prefrontal neuronal plasticity, particularly plasticity in the prelimbic and infralimbic cortex (Mikics et al., 2018).

In line with these findings, it was recently shown that there is a complex interplay between early stressors, plasma glucocorticoid levels, and decision-making at the level of the prefrontal cortex (Fan et al., 2014; Quevedo et al., 2017; Joëls et al., 2018). A key component of this interaction is HPA-axis functioning in adulthood, that depends to a large extent on early adversities (Raymond et al., 2017; Kaiser et al., 2018). Albeit the issue clearly needs further studies, findings obtained so far suggest that the link between early adversity, adult glucocorticoid deficits and abnormal aggression is established by epigenetic changes, and one of the major loci of these processes is the prefrontal cortex.

## Conclusions and hypothesis

Ample human evidence suggests that strong stressors downregulate the HPA-axis on the long run. Stressors that have this outcome largely coincide with those that have an opposite effect i.e., which upregulate the HPA-axis on the long run. The factors that underlie these opposite trajectories of HPA-axis responses are largely unknown at present, but disparate findings suggest that individual differences in stress responsiveness, and exposure to subsequent stressors may play an important role. The stress-induced decrease in glucocorticoid secretion capacities develop slowly. Low HPA-axis function on its turn was consistently associated with increases in aggressiveness and aggressive delinquency in humans. Findings suggest that the association is valid primarily for proactive, antisocial aggressiveness. Longitudinal studies indicate that the decrease in glucocorticoid secretion precedes the increase in aggressiveness by years, raising the possibility of a causal relationship. These human findings demonstrate the relevance of the relationship between glucocorticoid hypofunction and aggressiveness.

Hypothalamic mechanisms possibly underlying this relationship are difficult to study in humans, for which such mechanisms need to be investigated in animal models.

In rodents, the deliberate limitation of glucocorticoid secretion as well as conditions that on the long run decrease HPA-axis function result in increased aggression. This was observed in models of abnormal aggression, i.e., models that mimic the etiological factors of pathologic human aggression and where aggressive behavior strongly deviates from species-typical patterns. Recent findings suggest that an alternate neural route of aggression control mediates the effects of glucocorticoid deficits on aggression. Particularly, neural mechanisms of predatory aggression are activated in conditions associated with glucocorticoid deficits. Within this alternate mechanism, the lateral hypothalamus, especially the prefrontal cortex-lateral hypothalamic pathway seems to play an important role.

**Figure 3:**
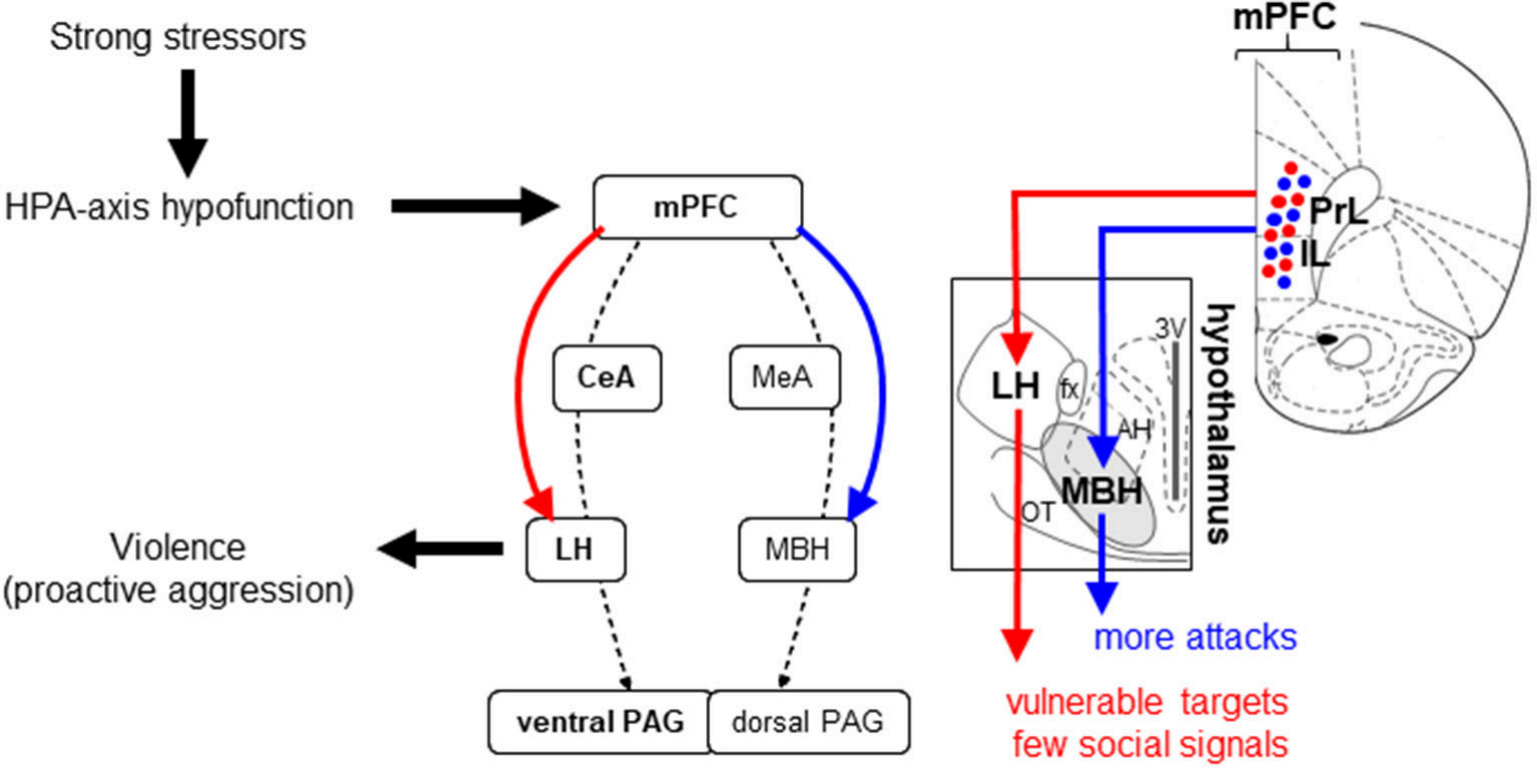
Brain mechanisms that mediate the effects of glucocorticoid deficits on aggression. Dashed arrows indicate hypothetical information flow based on earlier studies. Continuous arrows indicate the pathways discovered by Biro et al. (2018). The left hand panel is a simplified version of Figure 2. The arrows to the right and then to the left indicate the likely neural mechanism that link the hormonal to the behavioral event. The middle panel shows the “mixed” mechanism of glucocorticoid hypofunction-induced aggression complemented with two pathways that directly connect the medial prefrontal cortex to hypothalamic centers of aggression. The right hand panel shows on Paxinos and Watson slides (Paxinos and Watson, 1998) two subpopulations of medial prefrontal neurons, which project either to the lateral or mediobasal hypothalamus. The latter is an electrophysiologically defined area of the hypothalamus that covers several hypothalamic nuclei (Kruk, 1991). The optogenetic stimulation of axon terminals in the lateral hypothalamus increases the share of attacks on vulnerable targets that are poorly signaled socially. When stimulations are aimed at the mediobasal hypothalamus, the number of bites increases. Both effects are highly selective behaviorally (Biro et al., 2018). 3V, the third ventricle; AH, anterior hypothalamic nucleus; fx, fornix; IL, infralimbic cortex; OT, optic tract; PrL, prelimbic cortex. For other abbreviations, see Figure 1.

By corroborating human and animal findings, we hypothesize the following (Figure 3):

1. Strong stressors associated with subsequent stress exposure and high individual stress sensitivity decrease the glucocorticoid secretion capacity of the HPA-axis by a slowly developing process (“allostatic crash.”)
2. HPA-axis hypofunction alters the function of the aggression circuitry. One of the primary targets of this change is the prefrontal cortex. This process also develops slowly.
3. The ultimate consequence of brain alterations is that social challenges elicit the activation of brain mechanisms that originally control predatory aggression. This results in violent, harmful aggression.
4. The lateral hypothalamus plays a key role in eliciting violent forms of aggression. When the aggression circuitry is altered by glucocorticoid hypofunction, this brain region is activated by the prefrontal cortex and possibly by the central amygdala upon social challenge, and its activation leads to aggression patterns that are substantially more damaging than species-typical ones.

Ultimately, this hypothetical sequence of events explains how unfavorable (stressful) social conditions lead to endocrine and later to neural alterations that result in proneness to violence, which on its turn may restart the cycle of violence by creating social tensions that elicit hyporesponsive HPA-axes in others.

## Author contributions

The author confirms being the sole contributor of this work and approved it for publication.

### Conflict of interest statement

The author declares that the research was conducted in the absence of any commercial or financial relationships that could be construed as a potential conflict of interest.
